# The Treatment of Rectal Prolapse by the Submucous Injection of Paraffin

**Published:** 1904-10-01

**Authors:** 


### THE TREATMENT OF RECTAL PROLAPSE BY THE SUBMUCOUS INJECTION OF PARAFFIN.

The operation of injecting hard paraffin into the tissues to remedy slight deformities is now a well established practice. But the method hitherto has been chiefly used in the treatment of slight facial defects, such, for example, as sunken nose. Possibly the hesitation to make more frequent use of paraffin injections in other conditions has its origin in the one or two disastrous cases that have been recorded, such, for example, as Pfannenstiel's,[1] where pulmonary embolism occurred after the injection of 30 c.c. of paraffin for the cure of prolapse of the bladder. But it would appear, in the light of experience, that such accidents should be regarded as the results of imperfections in the technique and materials used rather than as dangers inseparable from the operation; and from the successes more recently recorded it seems likely that the scope of the procedure will be considerably extended. Mr. Arthur Burgess[2] reports the results of 18 cases in which he used paraffin injections to cure prolapse of the rectum, the patients' ages being from three to 48 years. The paraffin used melted at 111° F. The method he adopts is as follows: First, a syringe with rubber-covered barrel is warmed in a water bath at 120° F., then filled with sterilised paraffin at the same temperature and allowed to wait in the hot bath. The patient is anæsthetised and placed in the lithotomy position, the prolapse drawn down and its apex gently stretched into a triangular shape by three artery forceps attached to equidistant portions of the mucous membrane. The needle of the syringe is inserted, in turn, into the middle of the folds forming the sides of this triangle, and two or three c.c. of paraffin are introduced on each occasion. When this has become firm, the stiffened portion is pushed up and another lower part of the prolapse similarly treated, but the angles of this triangle are made to correspond with the lines of the preceding one. As a rule three such tiers are necessary. To prevent the paraffin entering a vein: (1) a blunt needle must be used, (2) the point must move easily in the submucous tissue, and (3) the fold of membrane should be raised with the forceps to form a good ridge. In the 18 cases, all severe, Mr. Burgess has had no case of recurrence of the prolapse. The advantages of this method over others, such as linear cauterisation, excision of folds of mucous membrane or of the prolapse, and colopexy, are its simplicity, the absence of risk to life, and the shortness of the after-treatment. The benefit is apparent immediately the paraffin has set, there is a greater probability of permanent cure, and even should this method fail, the patient's condition is no worse than before, and the procedure may be repeated.

[1] Stephen Paget, Brit. Med. Jour., 1903, p. 11. [2] Lancet, Sept. 10, 1904.

